# IRAM: virus capsid database and analysis resource

**DOI:** 10.1093/database/baz079

**Published:** 2019-07-18

**Authors:** Iman Almansour, Mazen Alhagri, Rahaf Alfares, Manal Alshehri, Razan Bakhashwain, Ahmed Maarouf

**Affiliations:** 1 Epidemic Diseases Department, Institute for Research and Medical Consultations, Imam Abdulrahman Bin Faisal University, P.O.Box 1982, Dammam 31441 Saudi Arabia; 2 Scientific and High Performance Computing Center, Deanship of Information and Communication Technology, Imam Abdulrahman Bin Faisal University, P.O.Box 1982, Dammam 31441 Saudi Arabia; 3 Department of Physics, Institute for Research and Medical Consultations, Imam Abdulrahman Bin Faisal University, P.O.Box 1982, Dammam 31441 Saudi Arabia

## Abstract

IRAM is an online, open access, comprehensive database and analysis resource for virus capsids. The database includes over 200 000 hierarchically organized capsid-associated nucleotide and amino acid sequences, as well as 193 capsids structures of high resolution (1–5 Å). Each capsid’s structure includes a data file for capsid domain (PDB), capsid symmetry unit (PDB) and capsid structure information (PSF); these contain capsid structural information that is necessary to run further computational studies. Physicochemical properties analysis is implemented for calculating capsid total charge at given radii and for calculating charge distributions. This resource includes BLASTn and BLASTp tools, which can be applied to compare nucleotide and amino acid sequences. The diverse functionality of IRAM is valuable to researchers because it integrates different aspects of virus capsids via a user-friendly interface. Such data are critical for studying capsid evolution and patterns of conservation. The IRAM database can also provide initial necessary information for the design of synthetic capsids for various biotechnological applications.

## Introduction

Capsids are monomeric protein shells that enclose viral nucleic acids and uniquely protect viruses from external conditions. A typical virus capsid comprises protein subunits that are grouped into morphological units called ‘capsomers’; these self-assemble to form the complete structure (1–3). In certain viruses, capsids are encoded by a single gene (4, 5), while in others, capsids are more complex and are generated from multiple polypeptide chains (6, 7). Capsids vary considerably in size,organization and symmetry (8–11).

Furthermore, virus capsids have naturally evolved to deliver their own genetic material into host cells with high efficiency. Studies have shown that capsid assembly is affected by the type of nucleic acid in the virus genome: in RNA viruses, capsids self-assemble around the viral nucleic acid; comparatively, in DNA viruses, capsid packaging occurs after capsid assembly (12–15). Due to their self-assembly properties, capsids have gained considerable attention in the gene delivery field as powerful carriers of nucleic acid vaccines and gene therapy (16–21). Similarly, capsids are being exploited for medical diagnostics, bioimaging and other bionanotechnological applications (22–25).

**Figure 1:**
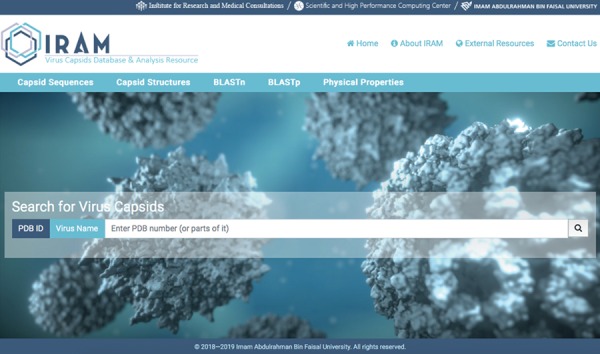
Snapshot of IRAM homepage.

Despite remarkable genetic diversity among viruses, evolutionary studies on capsids sequences have been largely focused on homologous viruses (26–35). Genomics and evolutionary studies among diverse viruses’ groups and families, however, have been limited (36–38). This is partially due to the unavailability of specialized databases on capsid sequences. Such data are crucial in understanding capsid evolution and patterns of conservation among diverse viral groups and host ranges. Furthermore, these data are necessary to help determine the relationship between sequence conservation and protein structure–function.

Since the discovery of virus structures by Casper and Klug (9, 39), x-ray crystallography and cryo-electron microscopy have provided an enormous body of information concerning capsid structure at or near the atomistic level. Despite these efforts, reports on the physical properties of capsids—such as charge distributions, assembly and disassembly—remain scarce, owing to their great complexity compared with other structural proteins (40–43). Thus, availability of specialized databases covering physical characteristics of individual capsid structure is crucial for accurate capsid modeling and for informing further biophysical simulation studies (Figure 1).

Presently, data on capsids are dispersed in different databases. The National Center for Biotechnology Information (NCBI) (https://www.ncbi.nlm.nih.gov) is a comprehensive database for all genes and protein sequences; the RCSB Protein Data Bank (https://www.rcsb.org) is a repository database for experimentally verified three-dimensional structural data of biological macromolecules; and the Virus Particle Explorer Database (http://viperdb.scripps.edu) is a specialized database for capsid PDB structures of diverse resolutions stored uniformly in *z*(2)-3–5-*x*(2) conventions (44). A comprehensive database for virus capsids nucleotide and amino acids sequences organized into taxonomic classification of viruses, as well as database for high-resolution capsids structural information, has yet to be developed. To address these needs, we present IRAM (https://iram.iau.edu.sa/), an open access online database of virus capsid information coupled with sequence- and structure-analytic capabilities. IRAM offers five major features:

(i) capsid sequences representing 15 different virus families;
(ii) capsid structural data files (PDB) at high resolution (1–5 Å);
(iii) capsid structural data files (PSF) containing physical attributes of capsids atoms;
(iv) capsid primary sequence alignments generated from BLASTn and BLASTp searches; and
(v) physicochemical properties calculator.

**Figure 2:**
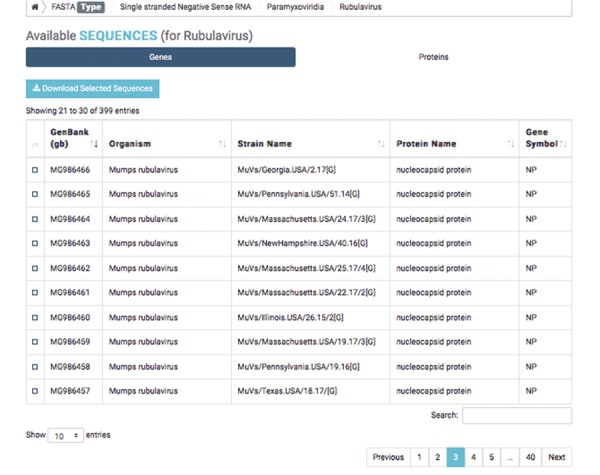
Detailed result of capsid nucleotide and amino acid sequences of a selected virus.

## Database architecture

### Capsid sequences

The first database includes over 200 000 capsid nucleotide and amino acid sequences, which have been manually curated from the NCBI and the National Institute of Allergy and Infectious Diseases’ Virus Pathogen Database and Analysis Resource (45). Database organization is based on the type of viral nucleic acid the capsids carry: single-stranded (ss) DNA, ssRNA, double-stranded (ds) DNA and dsRNA. Sequence taxonomy is based on the genomic classification of 15 virus families: *Arenaviridae*, *Bromoviridae*, *Bunyaviridae*, *Caliciviridae*, *Coronaviridae*, *Flaviviridae*, *Hepeviridae*, *Herpesviridae*, *Paramyxoviridae*, *Picornaviridae*, *Poxviridae*, *Reoviridae*, *Rhabdoviridae*, *Togaviridae* and *Virgaviridae*. Sequences are subcategorized according to genus and species. Users can download selected nucleotide or amino acid sequences; batch sequences affiliated with a given virus name can also be downloaded (Figure 2).

**Figure 3:**
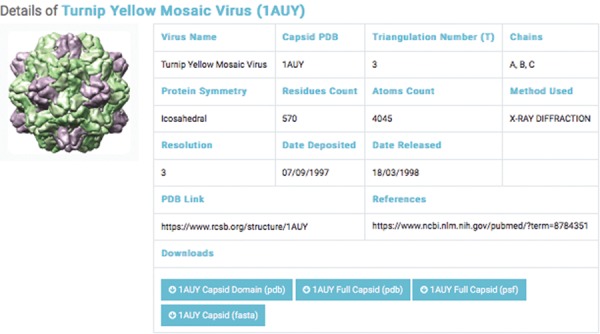
Details of a selected capsids structure. Capsid files: capsid domain PDB, full capsids PDB, full capsid PSF and capsid FASTA.

### Capsid structures

The second database includes structural data of 193 experimentally categorized capsid protein structures at high resolution (1–5 Å). Each capsid entry contains three capsid data files: capsid structural domains (PDB domain), retrieved from the PDB (46); capsid complete symmetry unit (Full capsid PDB), generated by Python-implemented Chimera (47); and capsid structure information (Full Capsid PSF), generated by CHARMM-implemented VMD (48) (Figure 3). Each PSF file generated includes topology and parameter subfiles containing data on the capsid’s atoms, bonds, angles, dihedrals, impropers (dihedral force terms used to maintain planarity) and cross terms. The PSF file contains necessary information for applying further molecular dynamics simulations—such as coarse-grained and all-atom molecular dynamics—to capsid structures.

We classified capsid structures based on resolution (1–5 Å). For each capsid structure entry, a link to a page was provided; the page contained the capsid’s name, PDB ID, triangular number, protein symmetry, residue counts, atom counts, method used, capsid PDB link and reference link. The interface allows the user to query by virus name or PDB ID (Figure 3).

## BLAST searches

BLASTn and BLASTp are provided as complementary tools for analyzing capsid nucleotide and amino acid sequences, respectively. The IRAM database BLAST module is built with NCBI BLAST+2.7.0, which allows users to compare sequences against the locally generated sequence database. The selection of specific BLASTn searches—such as blastn-short, dc-megablast and megablast, as well as blastp searches (blastp-short and blastp-fast)—is customized, and an E value output option is available. Results are displayed on the webpage and can also be downloaded.

## Physical properties calculator

Investigating the physicochemical properties of virus capsids may necessitate knowledge of their electronic charge distributions (49) and atomistic modeling (50,51). Charges of various atoms in the capsids of PDB-derived PSFs are treated as ‘point’ charges; however, because atomic charges are distributed throughout neighboring atomic positions, a point charge representation can lead to erroneous charge density calculations. This inaccuracy can be resolved by constructing a charge density function, *ρ*, defined at a general point ***r***, where ***r*** is measured from the center of the capsid. Therefore, we adopted a continuous and realistic charge density model by assuming that atomic charge takes a Gaussian form and that it is centered on each atom:

$$ \rho (r)=\frac{1}{{\left(4\pi a\right)}^{\left(3/2\right)}}{\sum}_{i=1}^N{q}_i{e}^{-{\left(\frac{r-{r}_i}{2a}\right)}^2}, $$

where ***r*** and *q_i_* are the location and charge of atom *i*, respectively; *a* is width (approximately 1.0 Å); and *N* is the number of atoms within the capsid. A reasonable estimate of *a* could be the van der Waals radius of an atom. Charges of various capsid atoms were taken from the PSFs, and auxiliary files were created for each capsid structure to facilitate various calculations. A charge density map can be plotted by using this density function, such as on the mid-plane of the capsid.

One advantage of using the constructed charge density function is that it involves an accurate calculation of the surface charge as a function of distance from the capsid’s center, as well as the charge contained within a certain volume of the capsid. This calculation is accomplished by integrating the charge density function over the desired region. Alternatively, this charge can also be obtained by a simple sum of the charges within the volume concerned. For example, to determine the charge contained between shells at 7 Å and at 10 Å, we use

$$ {Q}_{7\to 10}={\int}_7^{10} d r{\int}_0^{\pi } d\theta\ \mathit{\sin}\left(\theta \right){\int}_0^{2\pi } d\phi\ {r}^{\,2}\rho \left(r,\theta, \phi \right). $$

The surface charge density *σ(r)* is obtained from

$$ \sigma (r)={r}^{\,2}{\int}_0^{\pi } d\theta\ \mathit{\sin}\left(\theta \right){\int}_0^{2\pi } d\phi\ \rho \left(r,\theta, \phi \right). $$

**Figure 4:**
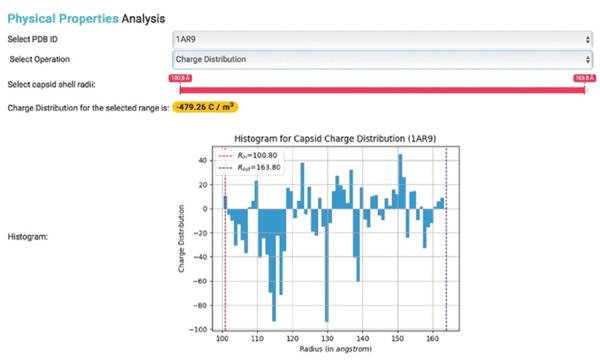
Physical properties analysis. Histogram showing the charge distribution of a selected capsid PDB ID, where R*_in_* represents inner capsid radii and R*_out_* represents outer capsid radii.

These built-in functions allow for the generation of various types of charge density plots for capsids in IRAM. Users with PDB IDs can also obtain corresponding PSFs and calculate capsid physical properties (Figure 4).

## Implementation

The web interface of IRAM was written in standard HTML/JavaScript/CSS using the Vue.JS framework at the front end. The back end was written in GO, building on the Go-Swagger OpenAPI framework. MongoDB was used for data storage. Docker was utilized to package and deploy all IRAM web application components. Data analytics and physical property computations of IRAM were implemented in Python and distributed using C++.

## Discussion and Conclusion

Here we introduced IRAM, an integrative platform and repository of over 200 000 capsid nucleotide and amino acid sequences and nearly 200 high-resolution capsid structures. The uniquely generated capsid structural information from PSF files can be used to study various biophysical molecular dynamics of capsids, such as assembly, disassembly and mechanical properties. We also implemented sequence analysis tools to aid researchers in exploring the evolutionary aspects of viral capsids and characterizing diverse host–virus interactions. Furthermore, we included a physical properties calculator of capsid charge distributions, which enables the study of capsids that are structurally similar but genetically divergent (42).

Engineering virus capsids is a vibrant area in synthetic biology, whereby capsids are exploited as drug and gene carriers. In this context, a specialized database, such as IRAM, is valuable for selecting capsid candidates based on sequence similarities and structural/physicochemical properties. For example, determining the inner and outer charges of capsids may contribute to the selection of capsids that are ideal for particular drug encapsulation. Furthermore, by integrating sequence and structural data into a single capsid database, researchers can effectively apply evolution-guided design of synthetic virus capsids.

We continue to add available capsid sequences to cover wider range of viruses for integration into the IRAM database. In addition, capsid structures will be updated as the relevant data become available. Future plans include capsid immune epitopes as well as expansion of physical properties analysis. Furthermore, we are developing a tool for introducing point mutations in wild-type capsids to create engineered virus models with unique properties; these can be studied for targeted delivery, altered tropism and evasion from antibody neutralization.

## Notes

**Database URL:** https://iram.iau.edu.sa
